# Meta-analysis reveals apolipoprotein ε4 confers higher susceptibility to Parkinson’s disease dementia in Asian populations

**DOI:** 10.3389/fnagi.2026.1737073

**Published:** 2026-03-10

**Authors:** Naseem Akhter, Ngoc Bao Phuong Ho, Ryan Nangreave, Saif Ahmad, Andrew F. Ducruet, Kanchan Bhatia

**Affiliations:** 1 Department of Translational Neuroscience, Barrow Neurological Institute, Phoenix, AZ, United States; 2 Arizona State University, Tempe, AZ, United States; 3 Department of Chemistry, Wofford College, Spartanburg, SC, United States; 4 Department of Translational Neuroscience, Barrow Neurological Institute, SJHMC, Dignity Health, Phoenix, AZ, United States; 5 Department of Neurosurgery, Barrow Neurological Institute, Phoenix, AZ, United States; 6 School of Mathematical and Natural Sciences, Arizona State University, Glendale, AZ, United States

**Keywords:** APOE polymorphism, apolipoprotein ε4, genetic susceptibility, meta-analysis, Parkinson’s disease dementia

## Abstract

Multiple studies show conflicting association between APOE polymorphisms and the risk of PDD, yielding inconsistent results. To elucidate, a meta-analysis was conducted using existing articles from Web of Science, PubMed, Cochrane, Google Scholar, Embase, WanFang, and CNKI databases, including case-control studies published up to January 31, 2025. A total of 27 studies (3,115 PD controls and 1,338 PDD cases) were included, with pooled Odds Ratio (ORs) and 95% confidence intervals (CIs) calculated using CMA, Biostat, United States. To assess APOE genotypes and PDD risk, three comparisons were examined: 5 genotypes vs. ε3/3, ε2+/ε4 + vs. ε3/3, and ε4 + vs. ε4−. The ε3/4 (OR = 1.56, 95% CI: 1.25–1.95); ε4 + vs. ε3/3 (OR = 1.52, 95% CI: 1.20–1.93) and ε4 + vs. ε4− (OR = 1.62, 95% CI: 1.39–1.90) genotypes were associated with an increased PDD risk, while ε2 + showed no significant effect (OR = 1.21, 95% CI: 0.88–1.65, *p* = 0.23). Carriers of ε4 + had a 1.52-fold higher risk compared to ε3/3, and the ε4 + vs. ε4 − comparison revealed a 1.62-fold greater dementia risk in ε4 + carriers. Subgroup analysis by ancestral region confirmed ε4 + as a significant risk factor for PDD across Asian, and Caucasians populations with higher susceptibility in Asian (OR = 1.98, 95% CI: 1.29–3.05) vs. Caucasian (OR = 1.48, 95% CI: 1.11–1.98) populations. Our findings suggest that ε3/4 and ε4/4 increase susceptibility to PDD, underscoring the need for further large-scale studies to validate these associations.

## Introduction

Parkinson’s disease (PD) is a progressive neurodegenerative disorder that primarily affects middle-aged and older adults, manifesting with both motor and nonmotor symptoms (Obeso et al., 2017). Classic motor features include resting tremor, rigidity, postural instability, and bradykinesia while nonmotor symptoms often involve olfactory dysfunction, sleep disorders, sensory disturbances, autonomic dysfunction, and cognitive decline. Among these, cognitive impairment represents one of the most debilitating complications, ranging from mild cognitive impairment to Parkinson’s disease dementia (PDD; Caviness et al., 2007).

Epidemiological studies estimate that dementia develops in approximately 25–30% of PD patients, with PDD accounting for 3–4% of all dementia cases (Aarsland et al., 2005). More recent investigations, however, suggest a lower or later incidence of dementia in PD, reporting 10-year probabilities of 9% based on clinical diagnosis, 15% using the MoCA, and 12% with the MDS-UPDRS cognitive score (Gallagher et al., 2024). These findings highlight the need for long-term prospective studies to more accurately characterize dementia risk in PD. Importantly, disease duration remains a strong determinant, as nearly 80% of patients surviving beyond two decades eventually develop cognitive decline (Halliday et al., 2008). This deterioration profoundly reduces quality of life, impairs social and occupational functioning, increases caregiver burden, and worsens overall survival. The progression of cognitive impairment in PD is heterogeneous, and its clinical characterization has been refined to distinguish PDD from dementia with Lewy bodies (DLB). Patients developing dementia more than 1 year after the onset of motor symptoms are classified as having PDD, whereas those presenting with cognitive impairment within 1 year of or before motor symptoms are diagnosed with DLB (McKeith, 2017). Nevertheless, accumulating evidence suggests that dementia onset in PD likely occurs along a continuum rather than as a strict categorical entity, reflecting complex and incompletely understood pathophysiological mechanisms. Genetic studies have consistently implicated the APOE ε4 allele as a risk factor for cognitive decline in PD, associated with an accelerated trajectory of cognitive deterioration (Bras et al., 2014; Guerreiro et al., 2018).

Despite these insights, the molecular pathways linking APOE ε4 to PDD remain unclear, and no disease-modifying treatments currently exist to halt or slow progression. Pathologically, PD is defined by insoluble *α*-synuclein (αSyn) aggregates within Lewy bodies (LBs) and Lewy neurites (LNs), which progressively spread across multiple brain regions, including the limbic system and neocortex in advanced stages (Halliday et al., 2008; Braak et al., 2003).

Notably, neuropathologic hallmarks of Alzheimer’s disease (AD), such as amyloid-*β* (Aβ) plaques and tau-containing neurofibrillary tangles, frequently coexist in PD-associated dementia (Irwin et al., 2013; Kotzbauer et al., 2012). Given the established role of APOE isoforms in AD pathogenesis (Kim et al., 2009; Holtzman et al., 2012), this overlap was long attributed to APOE-mediated modulation of cognition via comorbid AD pathology in PD (Irwin et al., 2017). However, emerging evidence indicates an independent contribution of APOE genotype to cognitive decline and Lewy pathology in PD, beyond its influence on AD-related changes (Tsuang et al., 2013; Dickson et al., 2018; Sabir et al., 2019).

Clinically, PDD presents with progressive deficits in attention, executive function, visuospatial ability, and memory, often accompanied by hallucinations, delusions, and affective disturbances (Emre et al., 2007). Pathological features include neurofibrillary tangles, LB accumulation, senile plaques, microvascular lesions, and argyrophilic inclusion bodies (Irwin et al., 2012; Del Tredici and Braak, 2013; Horvath et al., 2013; Halliday et al., 2014; Galvin et al., 2006). Established risk factors include advanced age, lower education, smoking, akinetic-rigid motor symptoms, mild cognitive impairment, REM sleep behavior disorder, and altered biomarkers such as reduced serum epidermal growth factor and uric acid (Xu et al., 2016). Genetic susceptibility further contributes to PDD risk, with several loci-including APOE, MAPT, SNCA, GBA, LRRK2, and COMT-implicated in disease vulnerability (Fagan and Pihlstrøm, 2017).

Among these, APOE has been most extensively studied due to its influence on dementia susceptibility. The APOE gene encodes three alleles—ε2, ε3, and ε4—forming six genotypes and three phenotypes: E2 (ε2/ε2, ε2/ε3), E3 (ε3/ε3), and E4 (ε3/ε4, ε2/ε4, ε4/ε4), with E3 considered the most prevalent and wild type (Yamazaki et al., 2019). These correspond to three protein isoforms—E2, E3, and E4—collectively termed APOE (Hauser et al., 2011; Mahley and Rall, 2000). APOE regulates cholesterol homeostasis, synaptic plasticity, neurogenesis, mitochondrial activity, tau phosphorylation, neuroinflammation, and *β*-amyloid metabolism (Tai et al., 2016; Grimaldi et al., 2024; Genin et al., 2011), exerting neuroprotective effects partly through oxidative stress reduction and extracellular signal-regulated kinase (ERK) signaling (Ghura et al., 2016; Gan et al., 2011).

Functionally, APOEε2 is generally neuroprotective, with longitudinal studies showing preservation of brain regions essential for daily functioning and episodic memory (Bonner-Jackson et al., 2012). By contrast, APOEε4 increases central nervous system vulnerability and is a major genetic risk factor for dementia (Kurz et al., 1996). Nevertheless, the association between APOE polymorphisms and PDD remains inconclusive. Some studies report no significant differences in APOE genotype distribution between PDD patients and controls (Kurz et al., 2009; Harhangi et al., 2000; Nicoletti et al., 2016), while others suggest that ε4 increases risk and ε2 may have variable effects (Monsell et al., 2014; Gomperts et al., 2013; Pang et al., 2018; Federoff et al., 2012). These discrepancies likely reflect differences in ethnicity, age, sex, diagnostic criteria, sample size, and methodology. A meta-analytic approach is therefore essential to integrate current evidence and clarify APOE’s role in PDD risk stratification.

Early studies exploring the association between APOEε4 polymorphisms and PDD risk yielded inconsistent results. A 2018 meta-analysis pooled data from 17 studies (820 PDD and 1,922 non-PDD cases) conducted prior to October 2017 (Pang et al., 2018). It examined three genotype contrasts (five genotypes vs. ε3/3, ε2+/ε4 + vs. ε3/3, and ε4 + vs. ε4-) and found that carriers of ε3/4 (OR 1.47, 95% CI 1.14–1.89) and ε4/4 (OR 2.93, 95% CI 1.20–7.14) had elevated risk of PDD, whereas ε2 + showed no significant effect. Overall, ε4 + carriers had a 1.72-fold greater dementia risk than ε4 − carriers, with consistent effects across Asian, European, and American populations. Although this work provided an important foundation, its modest sample size, reliance on studies published before 2017, and absence of stratification by population characteristics limited the strength of its conclusions. Since then, numerous additional studies have emerged, providing greater statistical power and the opportunity to explore ethnic differences in risk.

To address these gaps, the present meta-analysis incorporates 27 case–control studies (1,338 PDD cases and 3,115 PD controls) published up to January 31, 2025, sourced from multiple international databases. Using pooled ORs and 95% CIs, we re-examined these genotype contrasts and performed updated subgroup analyses by ancestral region. By leveraging this substantially larger and more recent dataset, our study provides a more definitive evaluation of APOE variants as genetic risk factors for PDD.

## Materials and methods

### Abstraction and data extraction

Current study duly followed the PRISMA guidelines for systematic reviews and meta-analyses (Liberati et al., 2009). A systematic and comprehensive search was conducted across PubMed, Web of Science, Cochrane, Google Scholar, Embase, WanFang, and CNKI databases to identify case–control and cohort studies examining the association between the APOE gene and the onset of PDD, published before January 31, 2025. To ensure totality, references from retrieved articles, conference proceedings, and gray literature were manually screened. The search strategy integrated subject and free terms, including PD, primary parkinsonism, paralysis agitans, Parkinson dementia complex, Apo E and its variants (APO-E, APO E, AD2, LPG, LDLCQ5), as well as cognitive impairment-related terms (dementia, cognitive disorders, cognitive defect, dementias, amentia). Additionally, methodological terms such as case–control study and cohort study were included. Key study characteristics were extracted: first author, publication date, study location, race, age, PD diagnostic criteria, dementia diagnostic criteria, study design, sample size, and genotype distributions of case and control groups. Two independent researchers conducted literature screening, quality assessment, and data extraction, with discrepancies resolved through consultation with a third researcher.

### Inclusion and exclusion criteria

This study focused on observational research examining the association between the APOE gene and the onset of PDD. Inclusion criteria mandated studies with a clinically or pathologically confirmed diagnosis of PD using established criteria (e.g., UK Brain Bank, Calne criteria, or China’s First National Symposium on Extrapyramidal Diseases). Additionally, studies had to report APOE genotyping, employ at least one dementia assessment method, and provide OR with 95% CI for case–control comparisons. Eligible studies included case–control or cohort designs, published in Chinese or English, with full-text availability or accessible data upon request. Exclusion criteria included studies that failed to align with the research focus, such as those excluding PD patients or investigating genes other than APOE. Studies were also excluded if they lacked explicit diagnostic criteria for PD, had incomplete genetic data, or failed to describe dementia assessment methods. Additionally, abstracts, literature reviews, case reports, and duplicate publications (where the most recent or comprehensive version was retained) were omitted. Studies with inaccessible full texts or unclear/incomplete sample data, even after contacting the authors, were also excluded.

### Statistical analysis

The relationship between the APOE gene and the onset of PDD was assessed by calculating ORs and 95% CIs for five genotypes (ε2/ε2, ε2/ε3, ε2/ε4, ε3/ε4, and ε4/ε4) compared to the ε3/3 genotype, as well as for ε2+/ε4 + vs. ε3/3 and ε4 + vs. ε4−. Between-study heterogeneity was evaluated using the chi-square-based Q-statistic test (Wu and Li, 1999). Depending on the significance of heterogeneity, either a random-effects or fixed-effects model was employed. A random-effects model was used when the *p*-value for heterogeneity was less than 0.05 (DerSimonian and Laird, 1986), while the fixed-effects model was applied in the absence of significant heterogeneity (Mantel and Haenszel, 1959). I^2^ statistics were used to quantify heterogeneity, with higher values indicating greater variability between studies (Higgins et al., 2003). Publication bias was assessed through Egger’s linear regression test and funnel plot asymmetry, considering a p-value less than 0.05 as indicative of significant bias (Egger et al., 1997). All analyses were conducted using Comprehensive Meta-Analysis (CMA) Version 4 software (Biostat, United States).

## Results

### Characteristics of eligible studies

**Figure 1:**
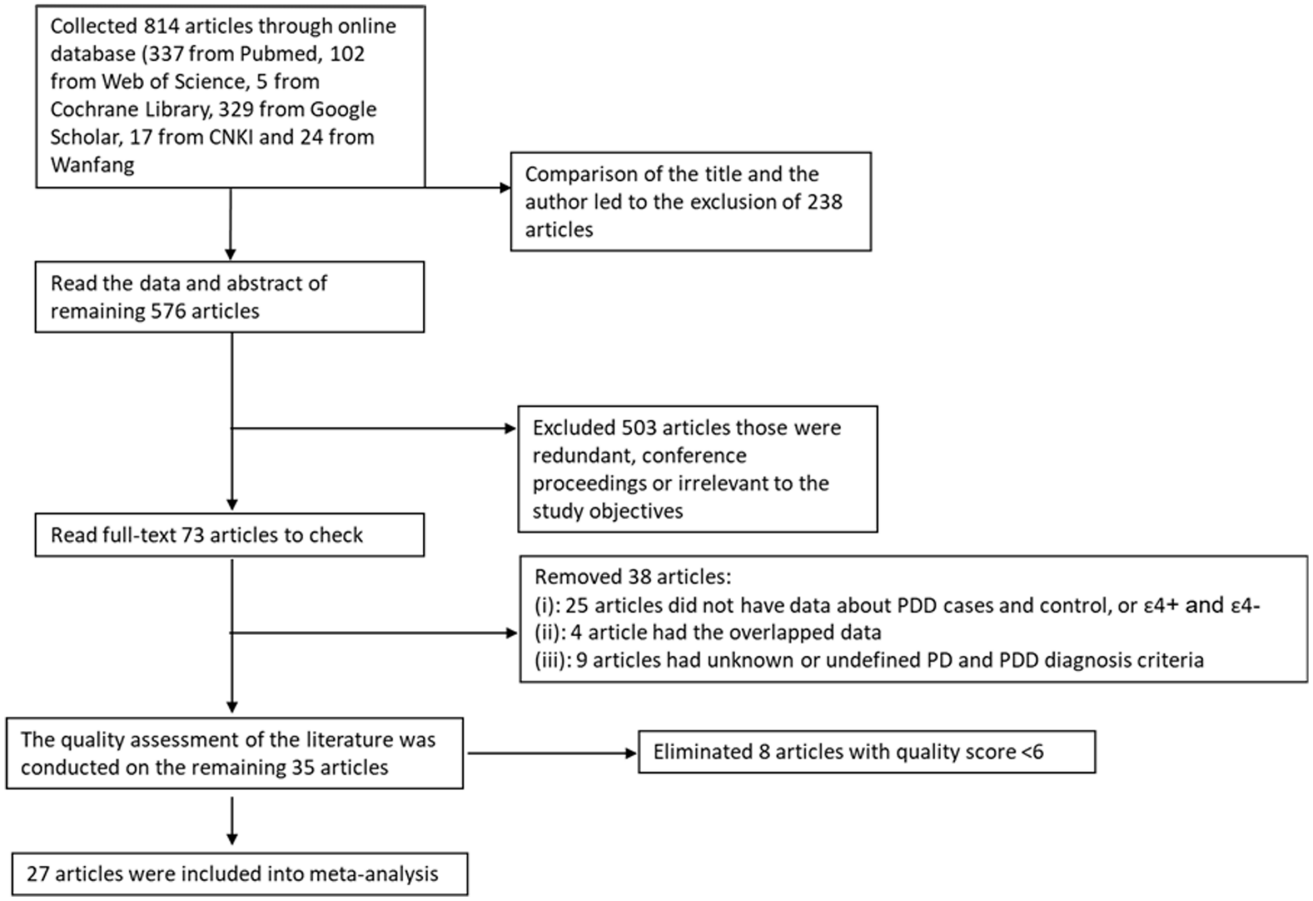
Flow diagram depicting the study selection process.

A comprehensive literature search across multiple online databases yielded a total of 814 articles. Initial screening based on titles and authors led to the exclusion of 238 articles. Abstract evaluations of the remaining 576 articles identified 503 as redundant, conference proceedings, or irrelevant to the study objectives, leaving 73 full-text articles for further assessment. Subsequent eligibility screening excluded 38 articles due to insufficient data on PDD cases and controls or ε4 + and ε4− variants (*n* = 25), overlapping datasets (*n* = 4), or undefined diagnostic criteria for PD and PDD (*n* = 9). Quality appraisal of the remaining 35 articles resulted in the removal of 8 studies with scores below 6 (Figure 1). Finally, our comprehensive meta-analysis incorporated 27 high-quality studies (6 Asian and 21 Caucasian cohorts), encompassing 1,338 cases and 3,115 controls, to evaluate the association between the ε4 allele of the APOE gene and dementia in PD (PDD) by comparing ε4 carriers (ε4+) and non-carriers (ε4−; Irwin et al., 2012; Nicoletti et al., 2016; Szwedo et al., 2022; Tunold, 2021; Mengel et al., 2016; Williams-Gray et al., 2009a; Tong, 2008; Wang, 2014; Ezquerra et al., 2008; Ma, 2007; Jasinska-Myga et al., 2007; Mollenhauer et al., 2006; Pankratz et al., 2006; Tröster et al., 2006; Blázquez et al., 2006; Camicioli et al., 2005; Zhou and Gui, 2004; Wang and Yang, 2001; Wakabayashi et al., 1998; Helisalmi et al., 1996; Egensperger et al., 1996; Morris, 1996; Koller, 1995; Martinoli et al., 1995; Han et al., 1994; Marder et al., 1994; Tables 1, 2).

**Table 1:** Major characteristics of the studies included in the meta-analysis.

S. No.	Author (s)	Reference number	Ethnicity	Study design	Cases	Controls	Newcastle-Ottawa score
1.	Szwedo et al. (2022)	49	Caucasian	Cohort	278	946	8
2.	Tunold (2021)	50	Caucasian	Cohort	80	71	7
3.	Mengel et al. (2016)	51	Caucasian	Case–control	72	375	6
4.	Nicoletti et al. (2016)	38	Caucasian	Case–control	25	24	8
5.	Wang (2014)	54	Asian	Case–control	97	157	8
6.	Irwin et al. (2012)	19	Caucasian	Case–control	89	42	7
7.	Williams-Gray et al. (2009a)	52	Caucasian	Cohort	19	88	7
8.	Tong (2008)	53	Asian	Case–control	20	67	7
9.	Ezquerra et al. (2008)	55	Caucasian	Case–control	86	138	7
10.	Ma (2007)	56	Asian	Case–control	81	133	8
11.	Jasinska-Myga et al. (2007)	57	Caucasian	Case–control	98	100	8
12.	Mollenhauer et al. (2006)	58	Caucasian	Case–control	60	23	7
13.	Pankratz et al. (2006)	59	Caucasian	Case–control	50	274	7
14.	Tröster et al. (2006)	60	Caucasian	Case–control	20	42	7
15.	Blázquez et al. (2006)	61	Caucasian	Case–control	31	245	7
16.	Camicioli et al. (2005)	62	Caucasian	Cohort	28	19	7
17.	Zhou and Gui (2004)	63	Asian	Case–control	15	36	6
18.	Wang and Yang (2001)	64	Asian	Case–control	11	40	6
19.	Harhangi et al. (2000)	37	Caucasian	Cohort	26	81	7
20.	Wakabayashi et al. (1998)	65	Asian	Cohort	12	10	7
21.	Helisalmi et al. (1996)	66	Caucasian	Cohort	8	15	6
22.	Egensperger et al. (1996)	67	Caucasian	Cohort	15	20	6
23.	Morris (1996)	68	Caucasian	Cohort	17	36	6
24.	Koller (1995)	69	Caucasian	Case–control	52	61	8
25.	Martinoli et al. (1995)	70	Caucasian	Case–control	18	10	7
26.	Han et al. (1994)	71	Caucasian	Case–control	8	5	6
27.	Marder et al. (1994)	72	Caucasian	Cohort	22	57	6

**Table 2:** Clinical characteristics of the studies included in the meta-analysis.

S. No.	Author (s)	Reference number	PDD diagnostic criteria	Dementia evaluation method	Sample capacity (experimental group and control group ≥ 50)	Patient source	Average age
1.	Szwedo et al. (2022)	49	UK Brain Bank	MMSE	No	Community	Average 69.1
2.	Tunold (2021)	50	UK Brain Bank	MMSE	Yes	Research center	PD61.3, PDD73.7
3.	Mengel et al. (2016)	51	UK Brain Bank	MDS-TFC	Yes	Research center	Average 66.7
4.	Nicoletti et al. (2016)	38	UK Brain Bank	MMSE	Yes	Unknown	Average 64.7
5.	Wang (2014)	54	UK Brain Bank	PDD diagnostic criteria	No	Hospital	PD65.20, PDD67.95
6.	Irwin et al. (2012)	19	UK Brain Bank	DSM	No	Research center	PD80, PDD79
7.	Williams-Gray et al. (2009b)	52	UK Brain Bank	DSM, MMSE	Yes	Community	Unknown
8.	Tong (2008)	53	UK Brain Bank	DSM, MMSE	Yes	Hospital	PD70.35, PDD75.44
9.	Ezquerra et al. (2008)	55	UK Brain Bank	PDD diagnostic criteria	No	Hospital	PD56, PDD58.3
10.	Ma (2007)	56	UK Brain Bank	DSM	No	Hospital	PD68.38, PDD69.72
11.	Jasinska-Myga et al. (2007)	57	UK Brain Bank	DSM, MMSE	Yes	Hospital	PD61.7, PDD71.4
12.	Mollenhauer et al. (2006)	58	UK Brain Bank	MMSE	No	Community	PD72, PDD72
13.	Pankratz et al. (2006)	59	UK Brain Bank	MMSE	No	Community	Average 60.9
14.	Tröster et al. (2006)	60	Calne criteria	DRS	Yes	Research center	Average 68.6
15.	Blázquez et al. (2006)	61	UK Brain Bank	MMSE	No	Hospital	Average 71.1
16.	Camicioli et al. (2005)	62	Pathology	DSM	No	Hospital	PD77.5, PDD78.1
17.	Zhou and Gui (2004)	63	Diagnostic criteria of National Symposium on Extrapyramidal Diseases in 1984	DSM	No	Hospital	Average 67.4
18.	Wang and Yang (2001)	64	Diagnostic criteria of National Symposium on Extrapyramidal Diseases in 1984	DSM	No	Hospital	PD66.13, PDD71.09
19.	Harhangi et al. (2000)	37	Calne criteria	DSM	No	Community	PD75.8, PDD82.1
20.	Wakabayashi et al. (1998)	65	Pathology	MSE	No	Community	PD65.1, PDD75*
21.	Helisalmi et al. (1996)	66	Pathology	MMSE	No	Unknown	PD71, PDD73
22.	Egensperger et al. (1996)	67	Pathology	PDD diagnostic criteria	No	Unknown	PD76, PDD75.8*
23.	Morris (1996)	68	Pathology	PDD diagnostic criteria	No	Unknown	Unknown
24.	Koller (1995)	69	Calne criteria	DRS	Yes	Research center	PD67.4, PDD74.7
25.	Martinoli et al. (1995)	70	Pathology	PDD diagnostic criteria	No	Mix	PD69, PDD68*
26.	Han et al. (1994)	71	Pathology	PDD diagnostic criteria	No	Research center	PD82, PDD75
27.	Marder et al. (1994)	72	Pathology	DSM	Yes	Community	PD69.9, PDD76.3

*Age at death; MMSE, Mini-Mental State Examination; MSE, Mental Status Examinations; MDS-TFC, Movement Disorder Society-Task Force Criteria; DSM:, Diagnostic and Statistical Manual; DRS, Mattis Dementia Rating Scale; PD, Parkinson’s disease without dementia; PDD, Parkinson’s disease with dementia.

### Association of all APOE genotypes with PDD risk

**Figure 2:**
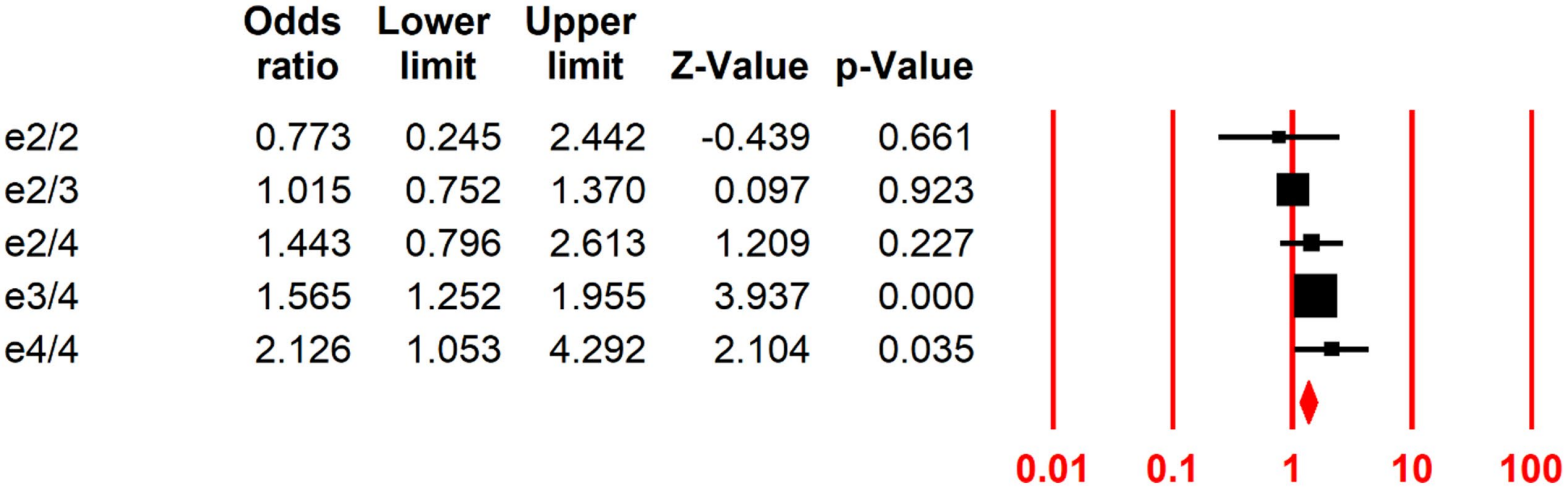
Risk of PDD across five *APOE* genotypes in comparison to the ε3/3 genotype (evaluated in 27 studies).

Among the studies analyzing all APOE genotypes, low genotype frequencies and zero event counts precluded the calculation of separate OR values. Consequently, ORs and 95% CIs were determined for five genotypes (ε2/ε2, ε2/ε3, ε2/ε4, ε3/ε4, and ε4/ε4) relative to ε3/ε3. PD patients carrying ε3/4 (OR 1.56, 95% CI 1.25–1.95) or ε4/4 (OR 2.12, 95% CI 1.05–4.29) exhibited a significantly higher risk of dementia relative to those with the ε3/3 genotype. In contrast, no significant risk difference was observed for ε2/ε2 (OR 0.77, 95% CI 0.24–2.44), ε2/ε3 (OR 1.01, 95% CI 0.75–1.37), or ε2/ε4 (OR 1.44, 95% CI 0.79–2.61; Figure 2; Table 3).

**Table 3:** Summary of the odds ratios (ORs) for all genetic comparison models.

Comparison models	ORs	CI (95%)		Z-value	*p*-value
		Lower limit	Upper limit		
Cumulative					
ɛ2 + vs. ɛ3/3	1.21	0.88	1.65	1.18	0.23
ɛ4 + vs. ɛ4−	1.62	1.39	1.90	6.18	0.00*
ɛ4 + vs. ɛ3/3	1.52	1.20	1.93	3.52	0.00*
Caucasian					
ɛ2 + vs. ɛ3/3	1.27	0.86	1.88	1.23	0.21
ɛ4 + vs. ɛ4−	1.59	1.34	1.88	5.46	0.00*
ɛ4 + vs. ɛ3/3	1.48	1.11	1.98	2.70	0.00*
Asian					
ɛ2 + vs. ɛ3/3	1.11	0.64	1.89	0.38	0.70
ɛ4 + vs. ɛ4−	1.85	1.23	2.80	2.96	0.00*
ɛ4 + vs. ɛ3/3	1.98	1.29	3.05	3.13	0.00*

OR, odd’s ratio; CI, confidence interval; *Statistically significant.

### Association of risk with PDD onset in ε2 + carriers

**Figure 3:**
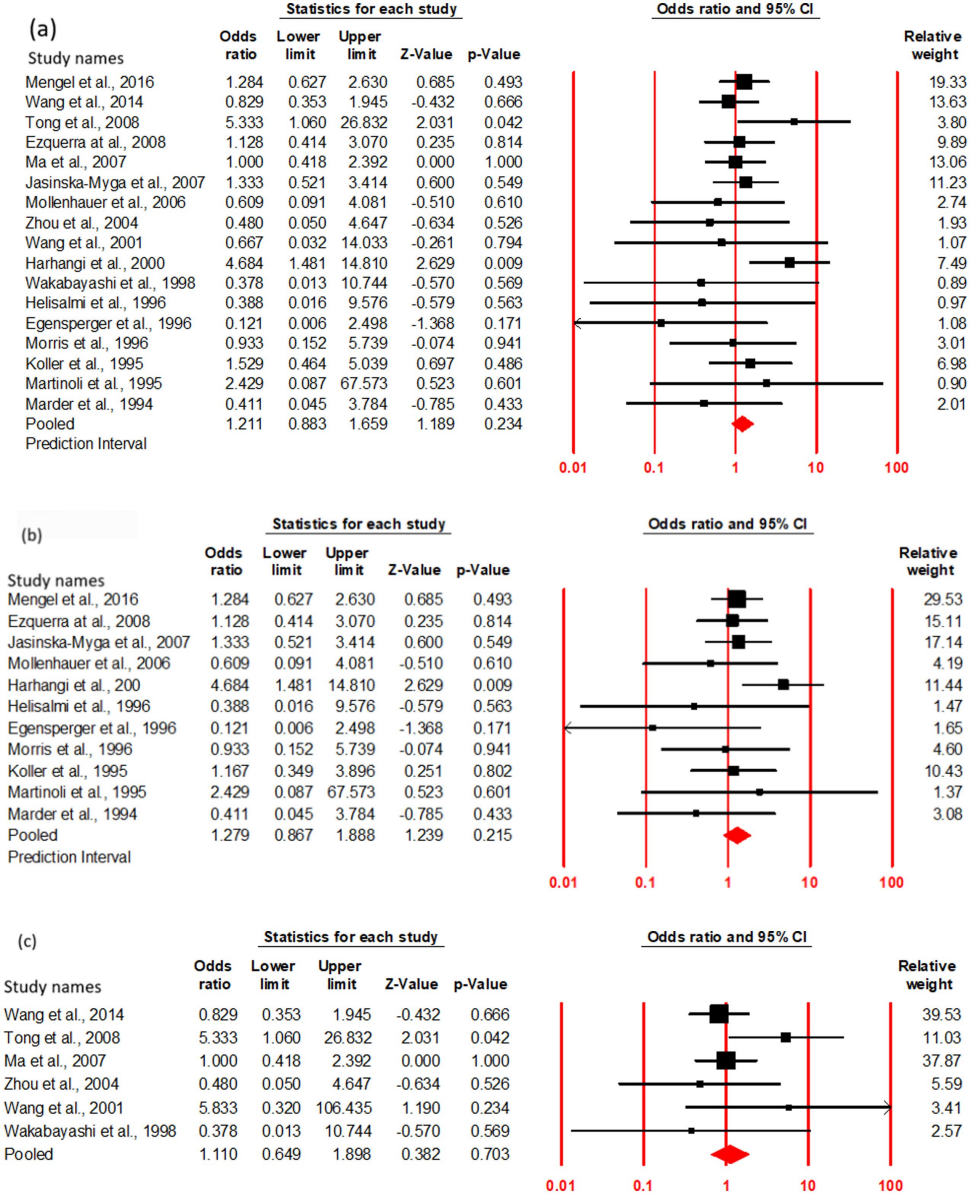
Forest plot of OR with 95% CI of PDD associated with the APOE ε2 + genotype. Black square represents the value of OR and the size of the square indicates the inverse proportion relative to its variance. Horizontal line is the 95% CI of OR. The studies are listed by year of publication. Forest plot with ORs on PDD risk associated with APOE ε2 + vs. ε3/3 genotype **(a)** Cumulative, **(b)** Caucasian, **(c)** Asian.

The OR and 95% CI for ε2 + vs. ε3/3 were analyzed across (a) cumulative (17studies), (b) Caucasian (11studies), and (c) Asian (6 studies) cohorts. No significant heterogeneity was observed (Q test, *p* > 0.10), justifying the use of a fixed-effect model (Table 4). The risk of PDD onset did not significantly differ between ε2 + and ε3/3 carriers in cumulative cohort (OR = 1.21, 95% CI: 0.88–1.65, *p* = 0.23). In order to assess any regional influence on PDD risk in ε2 + carriers, the Subgroup analysis based on geographic distribution categorized studies into Asian and Caucasian cohorts revealed no association of ε2 + genotype with an increased risk of PDD compared to ε3/3 carriers in both Caucasian (OR = 1.27, 95% CI: 0.86–1.88, *p* = 0.21) and Asian (OR = 1.11, 95% CI: 0.64–1.89, *p* = 0.70) populations (Figure 3; Table 3).

**Table 4:** Statistics to test publication bias and heterogeneity in the meta-analysis.

Comparison models	Egger’s regression analysis			Heterogeneity analysis				Model used
	Intercept	95% confidence interval	*p*-value (2-tailed)	Q-value	df (Q)	P_heterogeneity_	I^2^	
Cumulative								
ɛ2 + vs. ɛ3/3	−0.61	−1.70–0.48	0.25	15.33	16	0.50	0.00	Fixed
ɛ4 + vs. ɛ4−	0.73	−0.10–1.58	0.08	33.62	26	0.14	22.67	Fixed
ɛ4 + vs. ɛ3/3	0.67	−0.63–1.98	0.28	21.61	17	0.20	21.35	Fixed
Caucasian								
ɛ2 + vs. ɛ3/3	−0.94	−2.36–0.46	0.16	9.68	10	0.46	0.00	Fixed
ɛ4 + vs. ɛ4−	0.72	−0.27–1.72	0.14	27.33	20	0.12	26.83	Fixed
ɛ4 + vs. ɛ3/3	1.45	−0.25–3.16	0.08	14.49	10	0.15	31.02	Fixed
Asian								
ɛ2 + vs. ɛ3/3	0.56	−2.33–3.46	0.61	6.30	5	0.27	20.73	Fixed
ɛ4 + vs. ɛ4−	0.67	−2.47–3.82	0.58	5.82	5	0.32	14.13	Fixed
ɛ4 + vs. ɛ3/3	0.84	−2.00–3.69	0.45	5.38	5	0.37	7.19	Fixed

### Association of risk with PDD onset in ε4 + carriers vs. ε4– carriers

**Figure 4:**
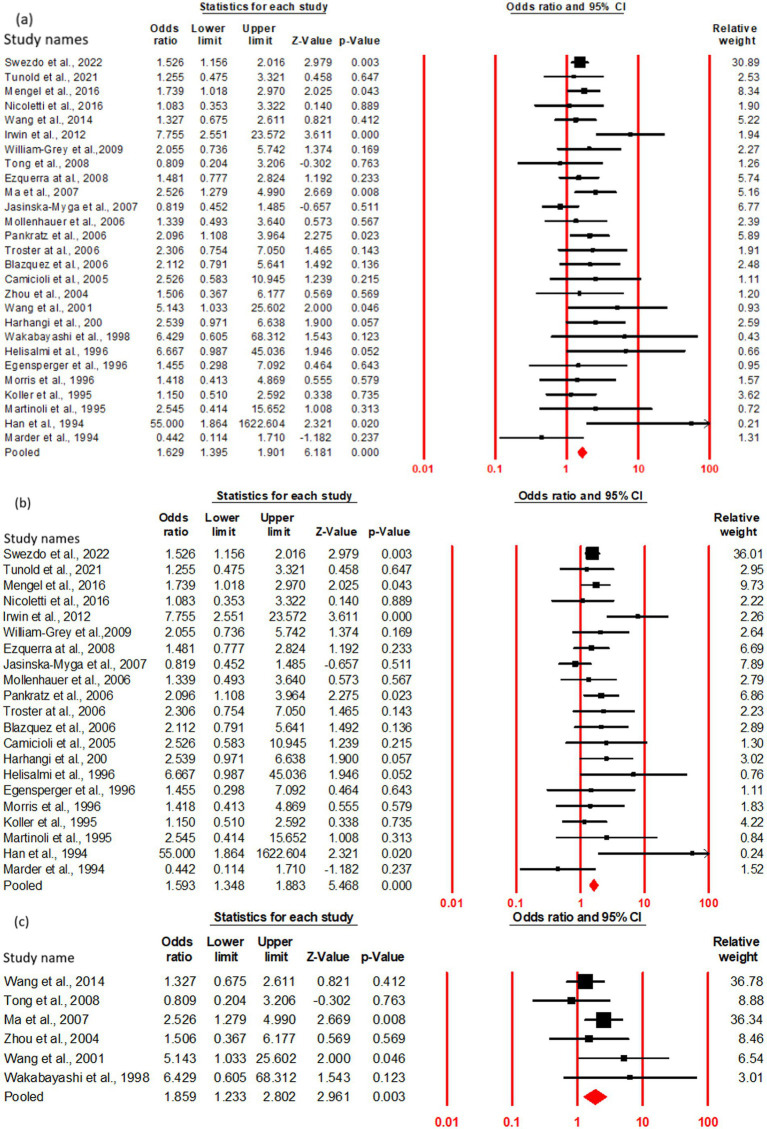
Forest plot of OR with 95% CI of PDD associated with the APOE ε4 + genotype. Black square represents the value of OR and the size of the square indicates the inverse proportion relative to its variance. Horizontal line is the 95% CI of OR. The studies are listed by year of publication. Forest plot with ORs on PDD risk associated with APOE ε4 + vs. ε4− genotype **(a)** Cumulative, **(b)** Caucasian **(c)** Asian.

The OR and 95% CI for ε4 + vs. ε4 − were analyzed across (a) cumulative (27 studies), (b) Caucasian (21 studies), and (c) Asian (6 studies) cohorts. The absence of significant heterogeneity (Q test, *p* > 0.10) justified the application of a fixed-effect model (Table 4). The results demonstrated a 1.62-fold increased risk of PDD in ε4 + PD patients compared to ε4 − carriers in the cumulative cohort (OR = 1.62, 95% CI: 1.39–1.90, *p* = 0.00). Subgroup analysis based on geographic distribution indicated a statistically significant association between the ε4 + genotype and elevated PDD risk in both Caucasian (OR = 1.59, 95% CI: 1.34–1.88, *p* = 0.00) and Asian (OR = 1.85, 95% CI: 1.23–2.80, *p* = 0.00) populations, suggesting a consistent genetic contribution to PDD susceptibility across ethnic groups (Figure 4; Table 3).

### Association of risk with PDD onset in ε4+

**Figure 5:**
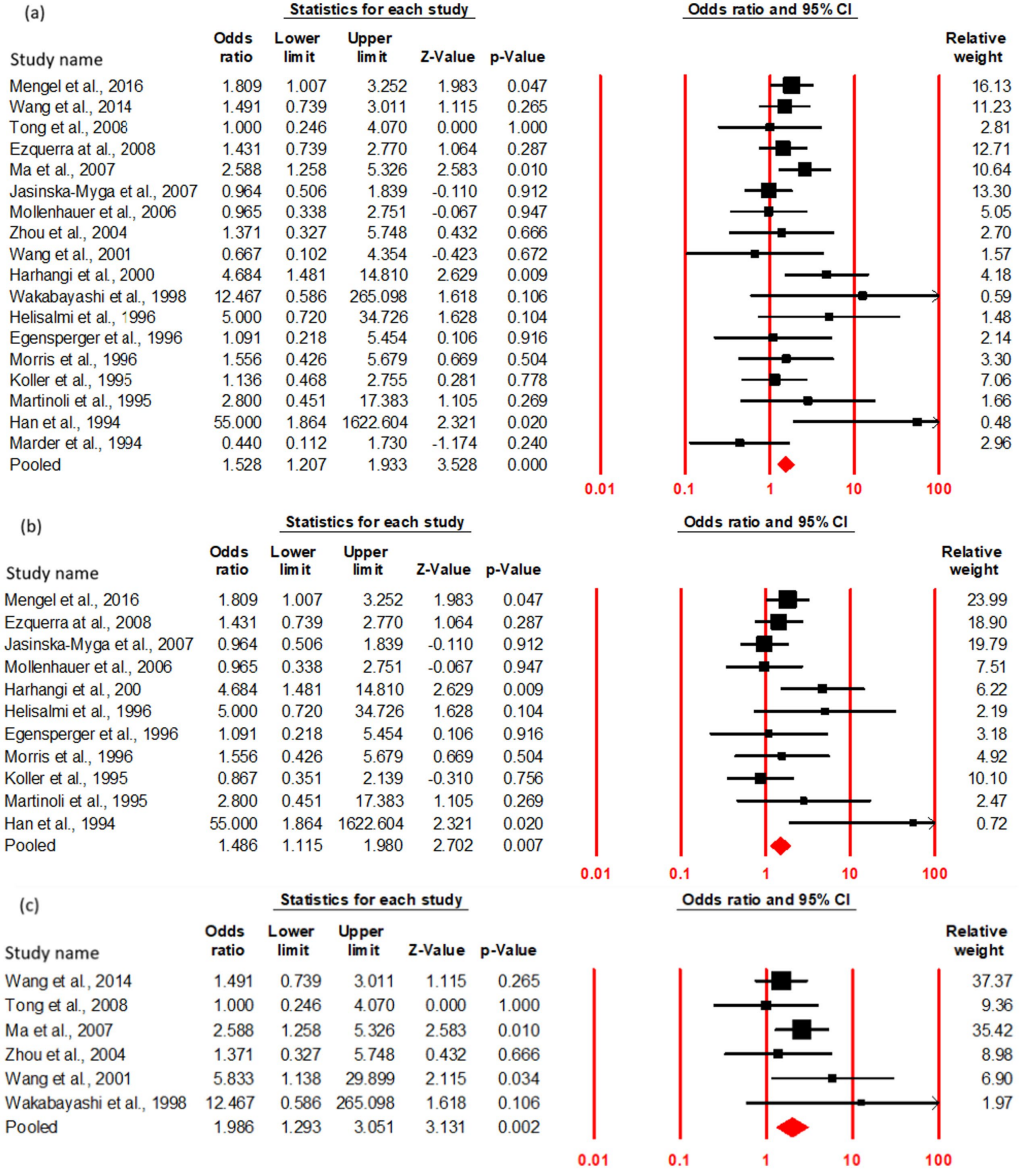
Forest plot of OR with 95% CI of PDD associated with the APOE ε4 + genotype. Black square represents the value of OR and the size of the square indicates the inverse proportion relative to its variance. Horizontal line is the 95% CI of OR. The studies are listed by year of publication. Forest plot with ORs on PDD risk associated with APOE ε4 + vs. ε3/3 genotype **(a)** Cumulative, **(b)** Caucasian **(c)** Asian.

The OR and 95% CI for ε4 + vs. ε3/3 were analyzed across (a) cumulative (18 studies), (b) Caucasian (11 studies), and (c) Asian (6 studies) cohorts. No significant heterogeneity was observed (Q test, *p* > 0.10), justifying the use of a fixed-effect model (Table 4). The cumulative cohort analysis demonstrated a 1.5-fold increased risk of PDD in ε4 + PD patients compared to those with the ε3/3 genotype (OR = 1.52, 95% CI: 1.20–1.93, p = 0.00). Subgroup analysis by geographic distribution further delineated this risk, revealing a statistically significant association between the ε4 + genotype and PDD in both Caucasian (OR = 1.48, 95% CI: 1.11–1.98, *p* = 0.00) and Asian (OR = 1.98, 95% CI: 1.29–3.05, *p* = 0.00) populations. Notably, the risk magnitude was higher in the Asian cohort, indicating a potential regional or ethnic susceptibility to ε4-associated neurodegeneration in PD (Figure 5; Table 3).

### Sensitivity analysis

**Figure 6:**
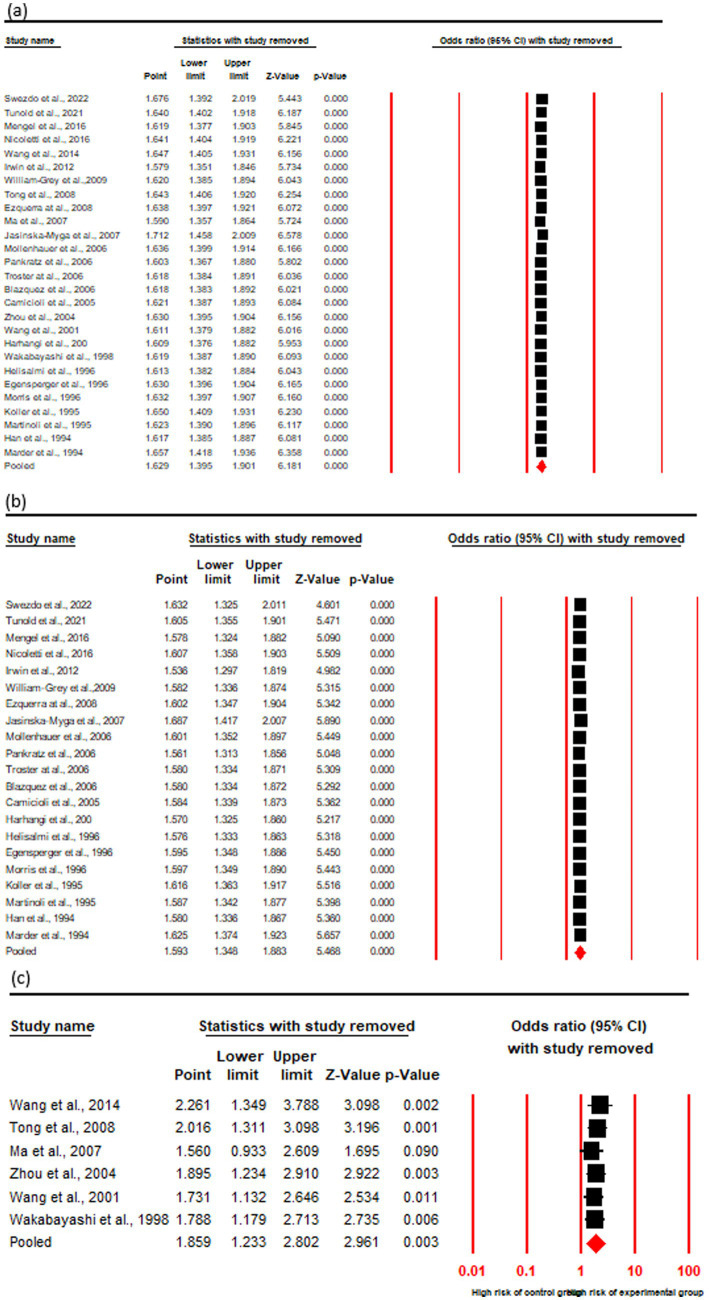
Sensitivity analysis by showing forest plot of OR with 95% CI of PDD associated with the *APOE* ε4 + genotype. Black square represents the value of OR and the size of the square indicates the inverse proportion relative to its variance. Horizontal line is the 95% CI of OR. The studies are listed by year of publication. Analysis results shown for *APOE* ε4 + vs. ε4− genotype **(a)** cumulative, **(b)** Caucasian **(c)** Asian.

**Figure 7:**
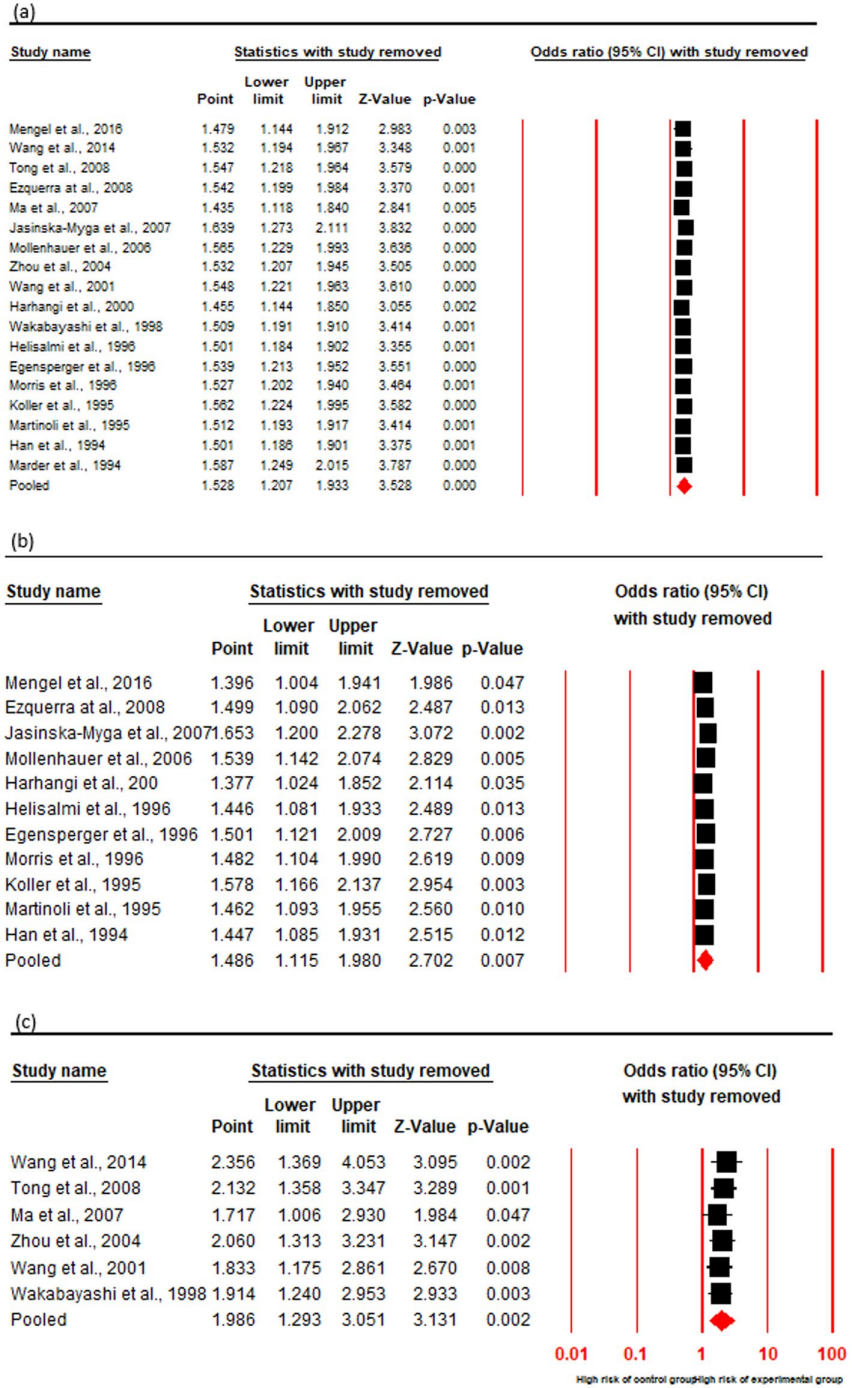
Sensitivity analysis by showing forest plot of OR with 95% CI of PDD associated with the APOE ε4 + genotype. Black square represents the value of OR and the size of the square indicates the inverse proportion relative to its variance. Horizontal line is the 95% CI of OR. The studies are listed by year of publication. Analysis results shown for APOE ε4 + vs. ε3/3 genotype: **(a)** Cumulative, **(b)** Caucasian, **(c)** Asian.

The stability of results was ascertained by assessing the influence of individual studies on the overall effect size, by recalculating iteratively, excluding one study at a time to determine its impact on the pooled effect size. This process identifies studies that may disproportionately affect the results, thereby assessing the reliability of the meta-analytic conclusions. In our analysis of the significant associations between ε4 + vs. ε3/3 and ε4 + vs. ε4−, sensitivity analyses revealed no substantial changes in the combined ORs, all of which remained statistically significant across all cohorts namely cumulative, Caucasian and Asian. Additionally, no individual studies were identified as contributing significant heterogeneity to the overall analysis (Figures 6, 7).

### Publication bias diagnosis

Egger’s test and Begg’s funnel plot were used to determine the evidence of publication bias among the included studies. Visual assessment of Funnel plots indicated symmetry of study distributions and thus an absence of potential biases. Further, Egger’s regression analysis revealed no evidence of publication bias across all cohorts—cumulative, Caucasian, and Asian. The intercept was greater than 0 with a non-significant *p*-value (*p* > 0.05), indicating the absence of significant asymmetry and supporting the conclusion that publication bias is not present (Table 4).

## Discussion

PD affects over 4 million individuals globally and is characterized by the degeneration of dopaminergic neurons (Chao et al., 2014). Genetic factors are recognized as contributors to PD pathogenesis (Lill, 2016), with mutations in genes including *APOE, SNCA*, *PARKIN*, *DJ-1*, *PINK1*, *MAPT*, and *LRRK2* associated with familial forms of the disease (Chao et al., 2014). Epidemiological evidence indicates that lipid metabolism and variations in lipid-metabolizing proteins or genes may influence neurodegenerative disease pathogenesis (Singh et al., 2014). Given the critical role of apolipoproteins and their receptors in lipid homeostasis, polymorphisms in APOE have been implicated as potential risk factors for Alzheimer’s disease (AD) and PD, with associations also observed in PDD (Tsuang et al., 2013; Tröster et al., 2006).

PDD is a major contributor to morbidity and caregiver burden in PD, yet no disease-modifying treatments or highly effective symptomatic therapies currently exist primarily due to its unelucidated molecular background. While cortical *α*-syn (αSyn) pathology correlates to some extent with cognitive impairment (Galvin et al., 2006; Braak et al., 2005; Hurtig et al., 2000), Lewy pathology alone does not fully explain the dementia phenotype. Biomarker analyses further suggest a complex pathological landscape in PDD (Halliday et al., 2008; Gifford, 2023; Gorges, 2019; Qi et al., 2024). A substantial proportion of individuals with PDD exhibit amyloid plaques and neurofibrillary tangles, implicating AD copathology in its progression (Irwin et al., 2017; Sengupta and Kayed, 2022). The strong genetic association between APOE ε4 and PDD is often linked to apoE4’s established role in Aβ aggregation (Holtzman et al., 2012; Huynh, 2017) and its connection to tau pathology (Shi et al., 2017). Although AD-related pathology—potentially APOE-dependent or independent—may significantly contribute to PDD, accumulating evidence also demonstrates a direct association between APOE genotype, Lewy body, neocortical Lewy neurite pathology, and dementia risk in PD. This suggests that APOE may influence αSyn pathophysiology independently of its interactions with Aβ or tau (Tsuang et al., 2013; Sabir et al., 2019).

We analyzed existing literature to elucidate the role of APOE polymorphisms in PDD risk. Given the well-established association between the APOE ε4 allele and AD risk, we aimed to determine whether this allele similarly correlates with dementia in PD (Michaelson, 2014). The role of the APOE ε4 allele in PDD remains inconsistent, few early studies identify APOE ε4 as a risk factor for dementia in PD (Monsell et al., 2014; Gomperts et al., 2013), while others find no association (Kurz et al., 2009; Harhangi et al., 2000; Nicoletti et al., 2016). These discrepancies may arise from differences in cognitive decline assessment methods, study population characteristics, sample sizes, and study designs. Notably, studies reporting negative findings often involve small PDD cohorts (fewer than 30 patients; Kurz et al., 2009; Nicoletti et al., 2016), whereas studies with positive findings typically include larger cohorts (more than 50 patients; Monsell et al., 2014; Gomperts et al., 2013). Additionally, two negative studies observed younger age at PD onset (in the 50s) among those experiencing rapid cognitive decline (Mengel et al., 2016; Ezquerra et al., 2008). This may reflect the slower annual cognitive decline in younger PD patients compared to older individuals, potentially misclassifying patients as having stable cognition in early disease stages—a limitation inherent to cross-sectional studies.

Our meta-analysis, which included 27 studies encompassing 1,338 patients with PDD and 3,115 with PD without dementia, reinforces the notion that APOE ε4 plays a critical role in cognitive decline among patients with PD. Across three key comparisons—individual genotypes vs. ε3/3, ε2+/ε4 + vs. ε3/3, and ε4 + vs. ε4 − —the ε3/4 and ε4/4 genotypes consistently conferred elevated risk, whereas the ε2 + genotype showed no significant association, with distributions comparable between PDD and control groups. Importantly, stratification into ε4 + and ε4 − carriers revealed a 1.52-fold increased risk of developing dementia among ε4 + individuals compared with ε3/3 carriers. These findings align with earlier reports highlighting the deleterious influence of APOE ε4 on neurodegenerative trajectories, while also confirming the neutral role of ε2 in this context. Moreover, the observation that the risk conferred by ε4 + varies ancestrally—with a nearly twofold increase in Asian populations vs. ~1.5-fold in Caucasians—suggests that ethnic or environmental modifiers may interact with APOE genotype to shape dementia risk. Collectively, these results underscore the importance of considering genetic background and population-specific factors when assessing vulnerability to PDD.

While our findings highlight the significant contribution of APOE ε4—particularly the ε3/4 and ε4/4 genotypes—to PDD risk across diverse populations, the biological mechanisms driving this association remain incompletely understood. The differential impact observed across ethnic groups further suggests that APOE genotype may interact with other genetic, environmental, or lifestyle factors to influence dementia susceptibility. Despite extensive research on APOE ε4 in dementia pathogenesis, the precise mechanism by which different APOE genotypes contribute to dementia development in PD remains unclear.

APOE ε4 influences dementia progression through multiple pathways. One major mechanism involves Aβ aggregation and clearance, where APOE modulates Aβ deposition via lipidation, facilitating its elimination through molecular chaperone interactions. However, APOE ε4 exhibits reduced clearance efficiency compared to APOE ε3/3, leading to Aβ accumulation, age-related pigment deposition, and cerebral amyloid angiopathy, all contributing to dementia (Hirsch-Reinshagen et al., 2009; Hanson et al., 2015). In addition to Aβ-related effects, APOE ε4 also promotes tau protein dysregulation.

While APOE ε3 and APOE ε2 interact with tau via cysteine residues to form stable complexes protecting against pathological phosphorylation, APOE ε4 has fewer cysteine residues, reducing its stabilizing capacity and promoting neurodegeneration (Huang, 2010). Additionally, APOE ε4 is linked to heightened neuroinflammation compared to APOE ε3, which may exacerbate neuronal injury and thereby contribute to dementia risk (Keene et al., 2011). Moreover, synaptic injury and repair are affected by APOE variants, with APOE ε4 carriers exhibiting reduced hippocampal dendritic density, contributing to cognitive decline (Chen, 2010).

Early meta-analyses consistently reported an elevated risk of dementia among PD patients carrying the APOE ε4 allele, although the magnitude of this effect varied across geographic regions (Pang et al., 2018; Huang et al., 2006; Sun et al., 2019). Subsequent longitudinal studies have reinforced this association, demonstrating that APOE ε4 is linked to accelerated cognitive decline, as assessed through global cognition screening tools (Schrag et al., 2017; Paul et al., 2016) and comprehensive cognitive batteries (Tropea et al., 2018; Morley et al., 2012). Genome-wide association studies have further substantiated this relationship, with rs429358—a variant tagging APOE ε4—emerging as the strongest genetic determinant of cognitive decline in PD (Tan, 2021). However, not all evidence has been consistent. For example, the CamPaIGN study, a well-characterized UK incident cohort, reported no significant association between APOE variants and either cognitive decline or dementia incidence over 3.5-, 5-, or even 10-year follow-up periods (Williams-Gray et al., 2009b; Williams-Gray et al., 2013), nor did it identify APOE status as a predictor of shorter time to dementia onset (Phongpreecha et al., 2020). These discrepancies underscore the complexity of APOE’s role in PD-related cognitive trajectories and suggest that additional genetic, environmental, and methodological factors may modulate its observed effects.

Several limitations of this meta-analysis should be acknowledged. First, the included studies employed heterogeneous diagnostic criteria for cognitive impairment, which may have introduced variability in outcome classification. Moreover, the onset and severity of cognitive decline are likely influenced by multiple factors—including age, education, smoking history, lifestyle, and additional dementia-associated genetic variants—that could not be systematically evaluated due to insufficient reporting in the original studies. This limitation also precluded meaningful subgroup analyses. Second, many of the individual studies were limited by small sample sizes, and several genotypes had zero event counts, restricting the calculation of ORs and preventing the performance of sensitivity analyses and heterogeneity testing. As a result, the robustness of certain genotype-specific estimates remains constrained, and risk prediction must rely on pooled data across studies. Finally, because our literature search was restricted to articles published in English and Chinese, the possibility of language bias cannot be excluded, and the generalization of the findings to other populations should be interpreted with caution.

Despite these limitations, an important strength of our work lies in the rigor of the current meta-analysis, which incorporated 27 high-quality studies—surpassing the scope of earlier analyses. By applying stricter inclusion and exclusion criteria, along with rigorous quality assessments, we ensured a more reliable synthesis of available evidence. Notably, recently published studies were incorporated, while those with suboptimal design, Newcastle–Ottawa Scale scores below 6, or insufficient diagnostic characterization of PD and PDD were deliberately excluded. This methodological refinement strengthens the robustness of our conclusions and enhances the confidence with which they can be interpreted.

While our findings consolidate evidence that APOE ε4 significantly increases the risk of dementia in PD, several gaps remain that warrant systematic investigation. First, harmonization of diagnostic criteria and cognitive assessment tools across cohorts is essential to reduce heterogeneity and improve comparability. Longitudinal studies with larger, multi-ethnic populations should be prioritized to capture the temporal dynamics of cognitive decline and clarify genotype–phenotype relationships.

Second, mechanistic studies are needed to disentangle the interplay between APOE variants, αSyn aggregation, Aβ deposition, and tau pathology. Employing multi-omics approaches—including genomics, transcriptomics, proteomics, and lipidomics—could provide deeper insights into how APOE ε4 modulates convergent neurodegenerative pathways. Integration of neuroimaging and fluid biomarkers will further aid in validating APOE’s role as a predictive and prognostic marker.

Third, stratification of patients by APOE genotype could enable precision-medicine approaches in both research and clinical settings. Clinical trials targeting lipid metabolism, neuroinflammation, or apoE4 structural correction may benefit from genotype-guided recruitment, improving sensitivity to treatment effects. Additionally, lifestyle and environmental modifiers such as diet, smoking, and physical activity should be systematically studied to identify modifiable risk factors that may mitigate APOE ε4–associated vulnerability.

Overall, our results are compatible with APOE ε4 contributing to PDD risk across ancestries, but the larger effect sizes seen in some populations may reflect population-specific factors (e.g., allele frequency, genetic background, gene-environment interactions, vascular comorbidity, or study heterogeneity); consequently, we cannot conclusively determine whether APOE ε4 acts via a single universal pathogenic mechanism or through ancestry-modulated pathways without further large, multi-ethnic and mechanistic studies.

Finally, translation to clinical care will require risk models that combine genetic, biomarker, and clinical data to identify individuals at greatest risk for rapid cognitive decline. Such predictive models could inform early intervention strategies, caregiver support planning, and the development of disease-modifying therapies aimed at slowing or preventing dementia in PD.

## Conclusion

This meta-analysis provides robust evidence that the APOE ε4 allele, particularly in ε3/4 and ε4/4 genotypes, is significantly associated with an increased risk of PDD, with stronger effects observed in Asian populations. In contrast, the ε2 allele appears to have no protective or risk-modifying role. These findings highlight APOE ε4 as a potential biomarker for identifying patients at higher risk of cognitive decline in PD. However, heterogeneity in study design and diagnostic criteria underscores the need for large, longitudinal studies to clarify underlying mechanisms and guide targeted interventions.

## Data Availability

The original contributions presented in the study are included in the article/Supplementary material, further inquiries can be directed to the corresponding authors.
