# Supplementary material for: Meta-analysis reveals apolipoprotein ε4 confers higher susceptibility to Parkinson’s disease dementia in Asian populations
Source: Front Aging Neurosci. 2026 Mar 10;18:1737073. doi: 10.3389/fnagi.2026.1737073 (PMC13008656; doi:10.3389/fnagi.2026.1737073)
Supplement: Supplementary file 1 [file Data_Sheet_1.pdf]

**Supplementary file 1. Full results of Egger's test and Begg's funnel plot for each comparison model**

Figure 1. Assessment of publication bias shown with Funnel plot in studies assaying odds of PDD associated with the **APOE** ε4+ genotype using effect size against precision, the inverse of standard error in APOE ε2+ versus ε3/3 genotype analysis namely (a) Cumulative, (b) Caucasian (c) Asian

Figure 2. Assessment of publication bias shown with Funnel plot in studies assaying odds of PDD associated with the **APOE** ε4+ genotype using effect size against precision, the inverse of standard error in APOE ε4+ versus ε4- genotype analysis namely (a) Cumulative, (b) Caucasian (c) Asian

Figure 3. Assessment of publication bias shown with Funnel plot in studies assaying odds of PDD associated with the **APOE** ε4+ genotype using effect size against precision, the inverse of standard error in APOE ε4+ versus ε3/3 genotype analysis namely (a) Cumulative, (b) Caucasian (c) Asian

1 (a)

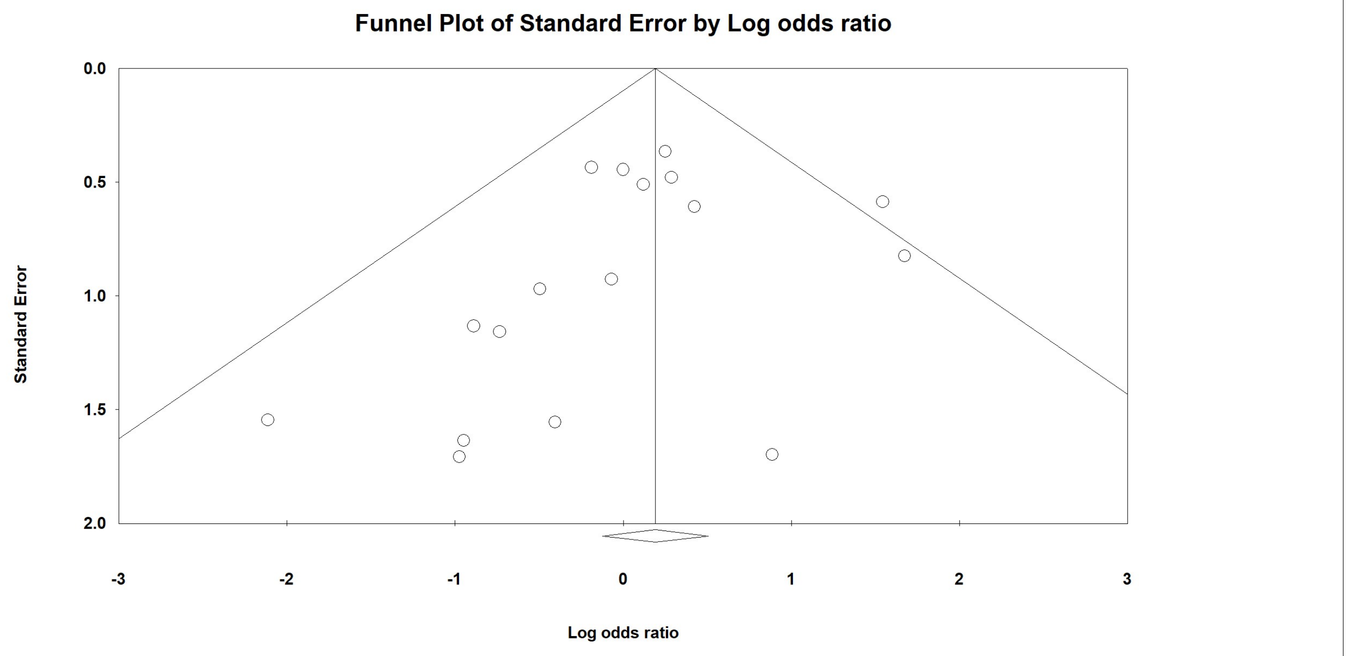

Funnel Plot of Standard Error by Log odds ratio
Standard Error
0.0
0.5
1.0
1.5
2.0
-3
-2
-1
0
1
2
3
Log odds ratio

## Egger's regression intercept

| | |
|---|---|
| Intercept | -0.61172 |
| Standard error | 0.51254 |
| 95% lower limit (2-tailed) | -1.70418 |
| 95% upper limit (2-tailed) | 0.48073 |
| t-value | 1.19352 |
| df | 15.00000 |
| P-value (1-tailed) | 0.12560 |
| P-value (2-tailed) | 0.25120 |

## Begg and Mazumdar rank correlation

| | |
|---|---|
| **Kendall's S statistic (P-Q)** | -22.00000 |

**Kendall's tau without continuity correction**

| | |
|---|---|
| Tau | -0.16176 |
| z-value for tau | 0.90624 |
| P-value (1-tailed) | 0.18240 |
| P-value (2-tailed) | 0.36481 |

**Kendall's tau with continuity correction**

| | |
|---|---|
| Tau | -0.15441 |
| z-value for tau | 0.86505 |
| P-value (1-tailed) | 0.19351 |
| P-value (2-tailed) | 0.38701 |

1 (b)

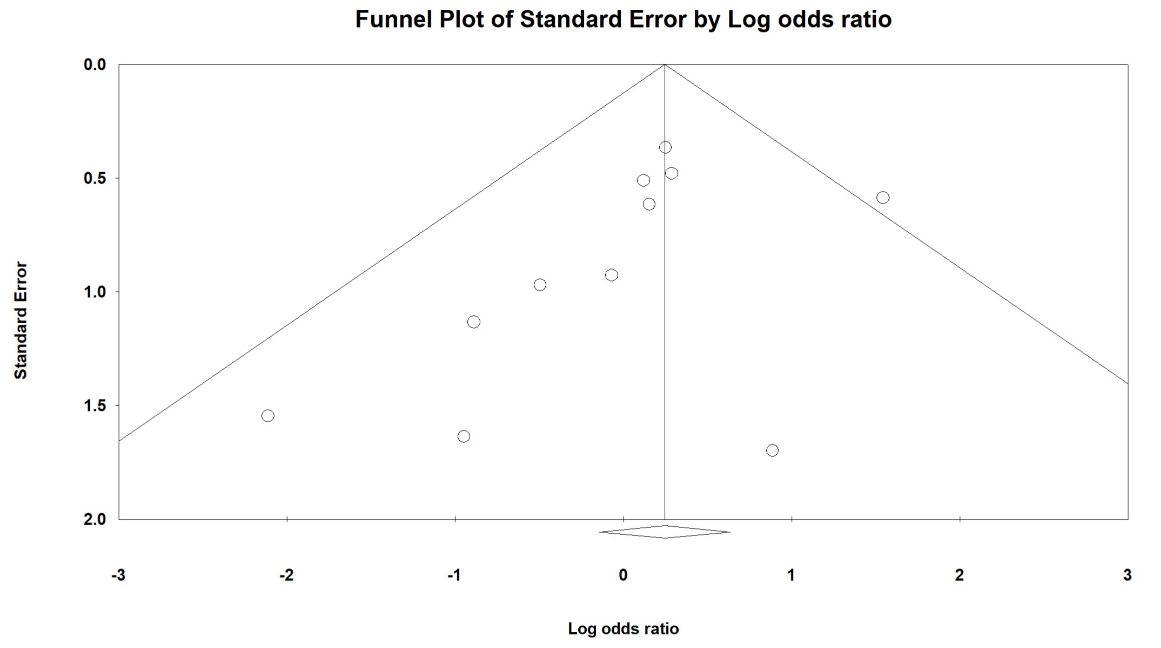
Funnel Plot of Standard Error by Log odds ratio
Standard Error
0.0
0.5
1.0
1.5
2.0
-3
-2
-1
0
1
2
3
Log odds ratio

## Egger's regression intercept

| | |
|---|---|
| Intercept | -0.94747 |
| Standard error | 0.62631 |
| 95% lower limit (2-tailed) | -2.36428 |
| 95% upper limit (2-tailed) | 0.46935 |
| t-value | 1.51277 |
| df | 9.00000 |
| P-value (1-tailed) | 0.08231 |
| P-value (2-tailed) | 0.16463 |

## Begg and Mazumdar rank correlation

| | |
|---|---|
| **Kendall's S statistic (P-Q)** | -21.00000 |

**Kendall's tau without continuity correction**

| | |
|---|---|
| Tau | -0.38182 |
| z-value for tau | 1.63485 |
| P-value (1-tailed) | 0.05104 |
| P-value (2-tailed) | 0.10208 |

**Kendall's tau with continuity correction**

| | |
|---|---|
| Tau | -0.36364 |
| z-value for tau | 1.55700 |
| P-value (1-tailed) | 0.05974 |
| P-value (2-tailed) | 0.11947 |

1 (c)

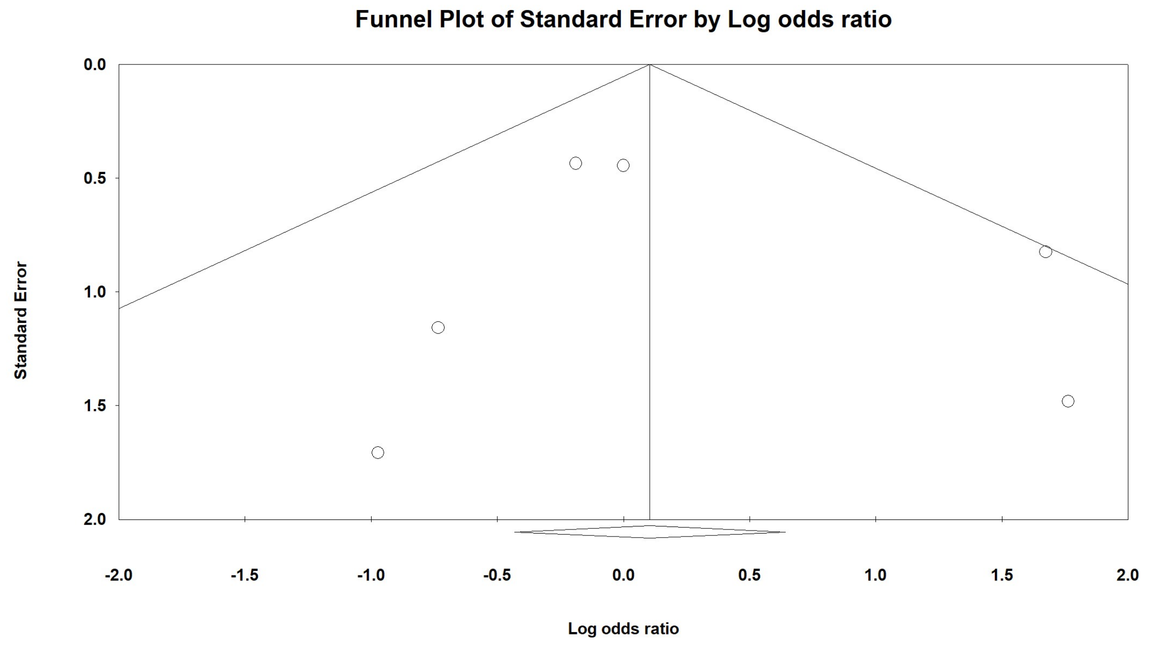

Funnel Plot of Standard Error by Log odds ratio
Standard Error
0.0
0.5
1.0
1.5
2.0
-2.0
-1.5
-1.0
-0.5
0.0
0.5
1.0
1.5
2.0
Log odds ratio

## Egger's regression intercept

| | |
|---|---|
| Intercept | 0.56671 |
| Standard error | 1.04523 |
| 95% lower limit (2-tailed) | -2.33531 |
| 95% upper limit (2-tailed) | 3.46873 |
| t-value | 0.54219 |
| df | 4.00000 |
| P-value (1-tailed) | 0.30824 |
| P-value (2-tailed) | 0.61648 |

## Begg and Mazumdar rank correlation

| | |
|---|---|
| **Kendall's S statistic (P-Q)** | 3.00000 |

**Kendall's tau without continuity correction**

| | |
|---|---|
| Tau | 0.20000 |
| z-value for tau | 0.56360 |
| P-value (1-tailed) | 0.28651 |
| P-value (2-tailed) | 0.57303 |

**Kendall's tau with continuity correction**

| | |
|---|---|
| Tau | 0.13333 |
| z-value for tau | 0.37573 |
| P-value (1-tailed) | 0.35356 |
| P-value (2-tailed) | 0.70711 |

2 (a)

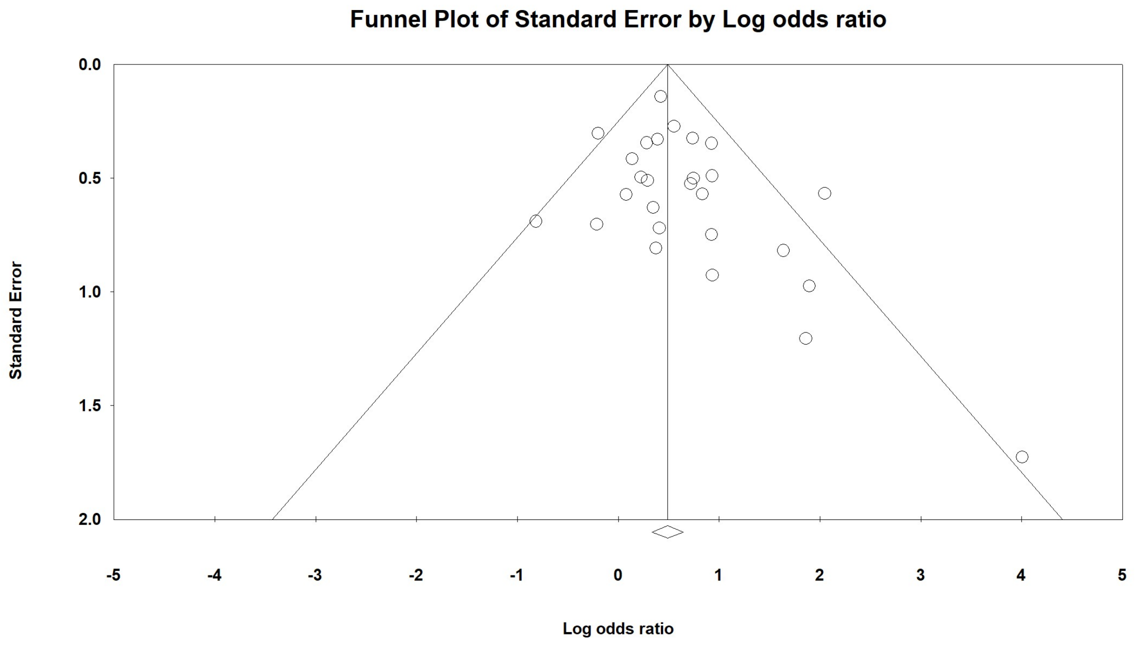
Funnel Plot of Standard Error by Log odds ratio
0.0
0.5
1.0
1.5
2.0
Standard Error
-5
-4
-3
-2
-1
0
1
2
3
4
5
Log odds ratio

## Egger's regression intercept

| | |
|---|---|
| Intercept | 0.73899 |
| Standard error | 0.41185 |
| 95% lower limit (2-tailed) | -0.10924 |
| 95% upper limit (2-tailed) | 1.58723 |
| t-value | 1.79431 |
| df | 25.00000 |
| P-value (1-tailed) | 0.04243 |
| P-value (2-tailed) | 0.08487 |

## Begg and Mazumdar rank correlation

| | |
|---|---|
| **Kendall's S statistic (P-Q)** | 95.00000 |

**Kendall's tau without continuity correction**

| | |
|---|---|
| Tau | 0.27066 |
| z-value for tau | 1.98046 |
| P-value (1-tailed) | 0.02383 |
| P-value (2-tailed) | 0.04765 |

**Kendall's tau with continuity correction**

| | |
|---|---|
| Tau | 0.26781 |
| z-value for tau | 1.95961 |
| P-value (1-tailed) | 0.02502 |
| P-value (2-tailed) | 0.05004 |

2 (b)

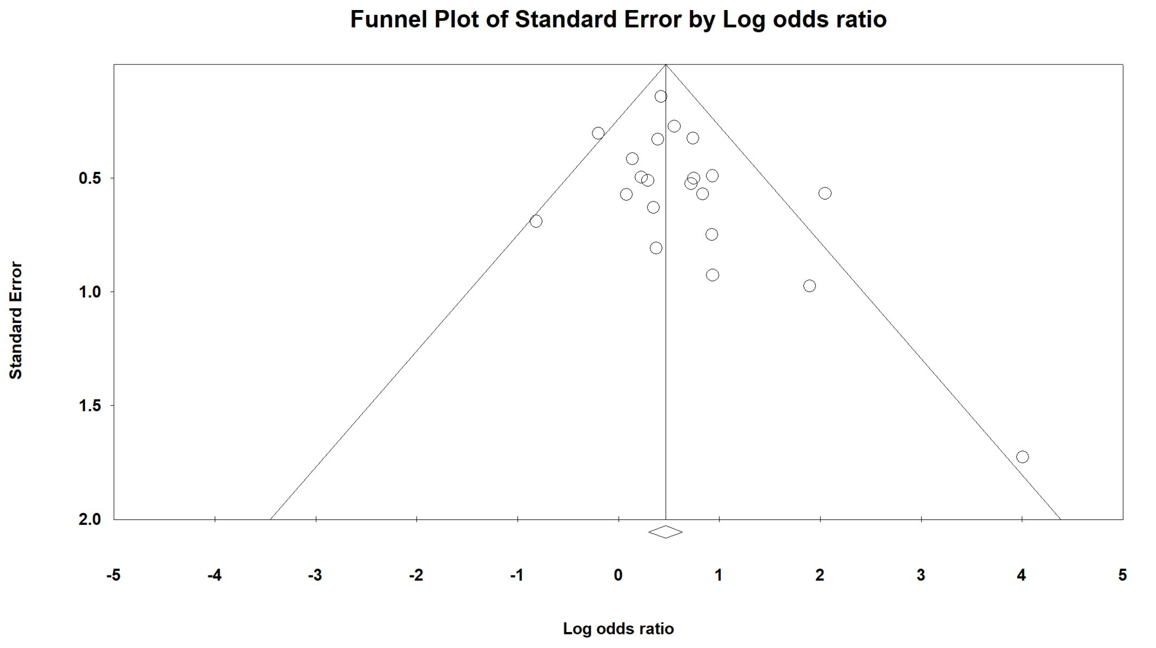

Funnel Plot of Standard Error by Log odds ratio
Standard Error
0.5
1.0
1.5
2.0
-5
-4
-3
-2
-1
0
1
2
3
4
5
Log odds ratio

## Egger's regression intercept

| | |
|---|---|
| Intercept | 0.72443 |
| Standard error | 0.47686 |
| 95% lower limit (2-tailed) | -0.27365 |
| 95% upper limit (2-tailed) | 1.72252 |
| t-value | 1.51916 |
| df | 19.00000 |
| P-value (1-tailed) | 0.07259 |
| P-value (2-tailed) | 0.14519 |

## Begg and Mazumdar rank correlation

| | |
|---|---|
| **Kendall's S statistic (P-Q)** | 54.00000 |

**Kendall's tau without continuity correction**

| | |
|---|---|
| Tau | 0.25714 |
| z-value for tau | 1.63063 |
| P-value (1-tailed) | 0.05148 |
| P-value (2-tailed) | 0.10297 |

**Kendall's tau with continuity correction**

| | |
|---|---|
| Tau | 0.25238 |
| z-value for tau | 1.60044 |
| P-value (1-tailed) | 0.05475 |
| P-value (2-tailed) | 0.10950 |

2 (c)

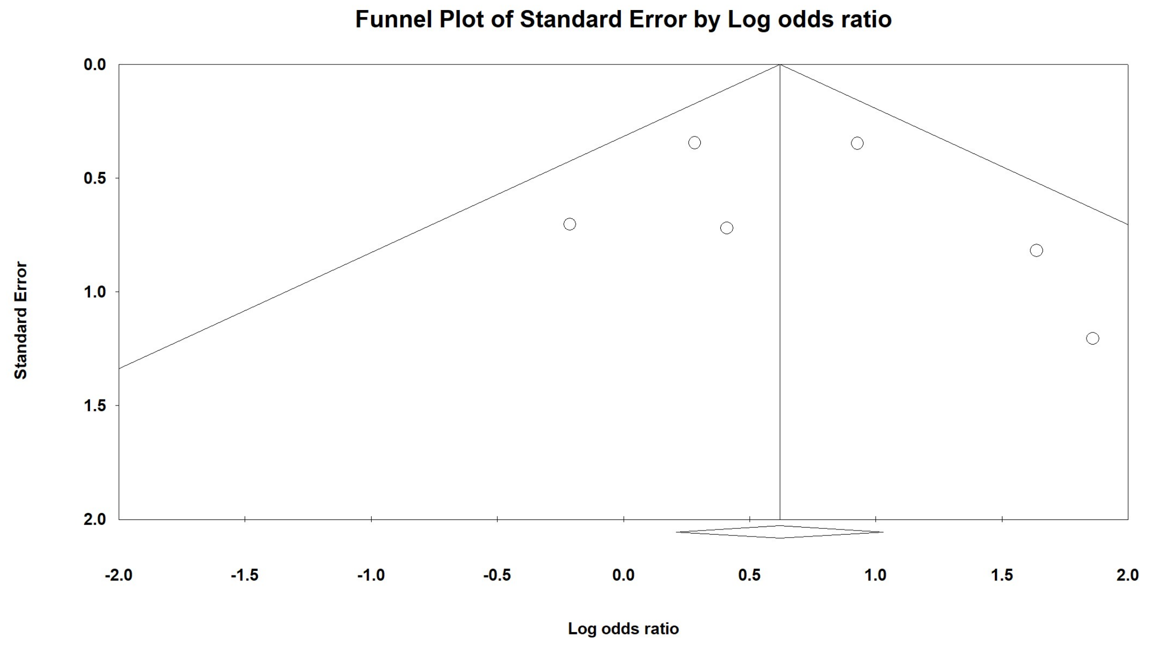
Funnel Plot of Standard Error by Log odds ratio
Standard Error
0.0
0.5
1.0
1.5
2.0
-2.0
-1.5
-1.0
-0.5
0.0
0.5
1.0
1.5
2.0
Log odds ratio

## Egger's regression intercept

| | |
|---|---|
| Intercept | 0.67788 |
| Standard error | 1.13393 |
| 95% lower limit (2-tailed) | -2.47043 |
| 95% upper limit (2-tailed) | 3.82619 |
| t-value | 0.59781 |
| df | 4.00000 |
| P-value (1-tailed) | 0.29108 |
| P-value (2-tailed) | 0.58217 |

## Begg and Mazumdar rank correlation

| | |
|---|---|
| **Kendall's S statistic (P-Q)** | 5.00000 |

**Kendall's tau without continuity correction**

| | |
|---|---|
| Tau | 0.33333 |
| z-value for tau | 0.93934 |
| P-value (1-tailed) | 0.17378 |
| P-value (2-tailed) | 0.34756 |

**Kendall's tau with continuity correction**

| | |
|---|---|
| Tau | 0.26667 |
| z-value for tau | 0.75147 |
| P-value (1-tailed) | 0.22619 |
| P-value (2-tailed) | 0.45237 |

3 (a)

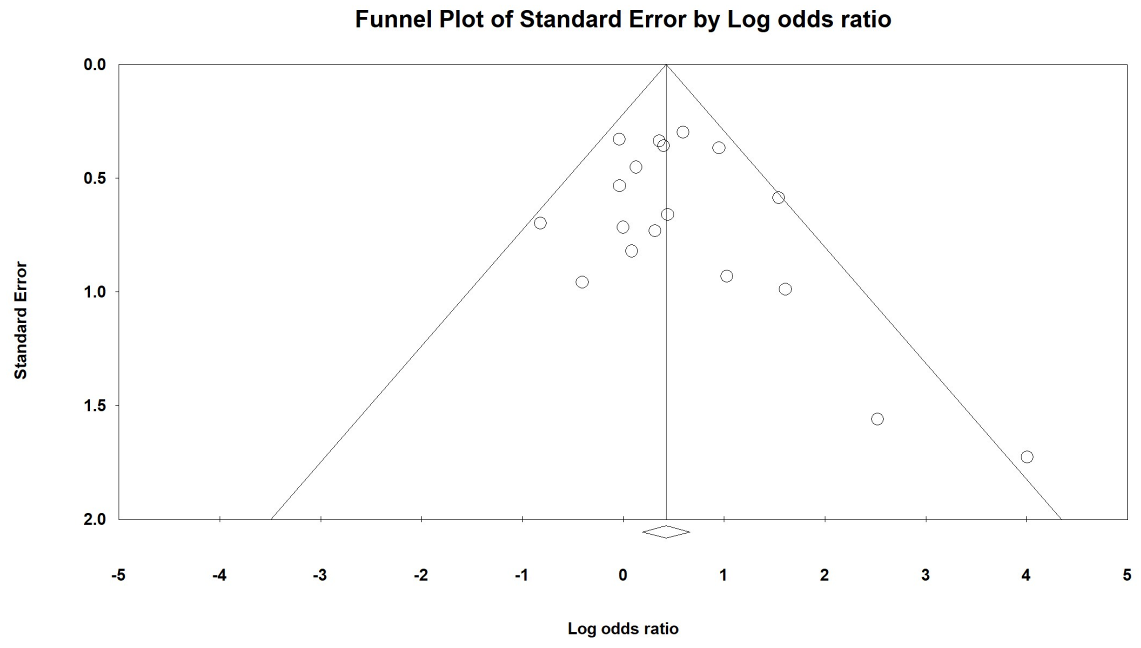
Funnel Plot of Standard Error by Log odds ratio
Standard Error
0.0
0.5
1.0
1.5
2.0
-5
-4
-3
-2
-1
0
1
2
3
4
5
Log odds ratio

## Egger's regression intercept

| | |
|---|---|
| Intercept | 0.67579 |
| Standard error | 0.61666 |
| 95% lower limit (2-tailed) | -0.63147 |
| 95% upper limit (2-tailed) | 1.98305 |
| t-value | 1.09588 |
| df | 16.00000 |
| P-value (1-tailed) | 0.14468 |
| P-value (2-tailed) | 0.28935 |

## Begg and Mazumdar rank correlation

| | |
|---|---|
| **Kendall's S statistic (P-Q)** | 35.00000 |

**Kendall's tau without continuity correction**

| | |
|---|---|
| Tau | 0.22876 |
| z-value for tau | 1.32572 |
| P-value (1-tailed) | 0.09247 |
| P-value (2-tailed) | 0.18493 |

**Kendall's tau with continuity correction**

| | |
|---|---|
| Tau | 0.22222 |
| z-value for tau | 1.28784 |
| P-value (1-tailed) | 0.09890 |
| P-value (2-tailed) | 0.19780 |

3 (b)

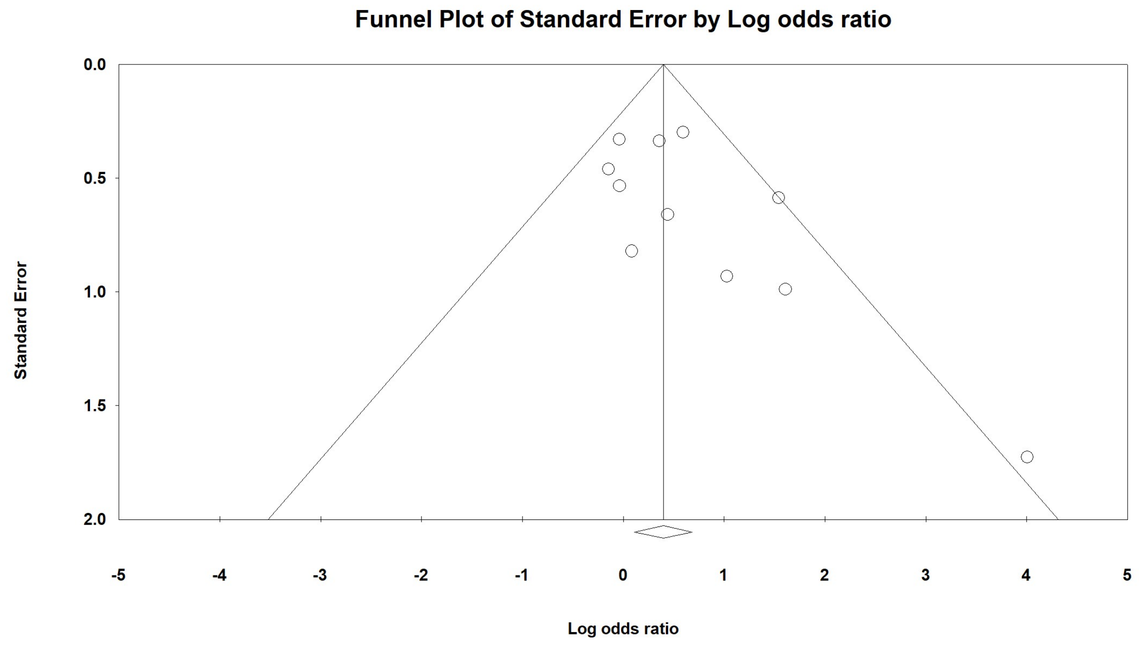
Funnel Plot of Standard Error by Log odds ratio
Standard Error
0.0
0.5
1.0
1.5
2.0
-5
-4
-3
-2
-1
0
1
2
3
4
5
Log odds ratio

## Egger's regression intercept

| | |
|---|---|
| Intercept | 1.45755 |
| Standard error | 0.75629 |
| 95% lower limit (2-tailed) | -0.25329 |
| 95% upper limit (2-tailed) | 3.16839 |
| t-value | 1.92724 |
| df | 9.00000 |
| P-value (1-tailed) | 0.04303 |
| P-value (2-tailed) | 0.08605 |

## Begg and Mazumdar rank correlation

| | |
|---|---|
| **Kendall's S statistic (P-Q)** | 25.00000 |

### Kendall's tau without continuity correction

| | |
|---|---|
| Tau | 0.45455 |
| z-value for tau | 1.94625 |
| P-value (1-tailed) | 0.02581 |
| P-value (2-tailed) | 0.05163 |

### Kendall's tau with continuity correction

| | |
|---|---|
| Tau | 0.43636 |
| z-value for tau | 1.86840 |
| P-value (1-tailed) | 0.03085 |
| P-value (2-tailed) | 0.06171 |

3 (c)

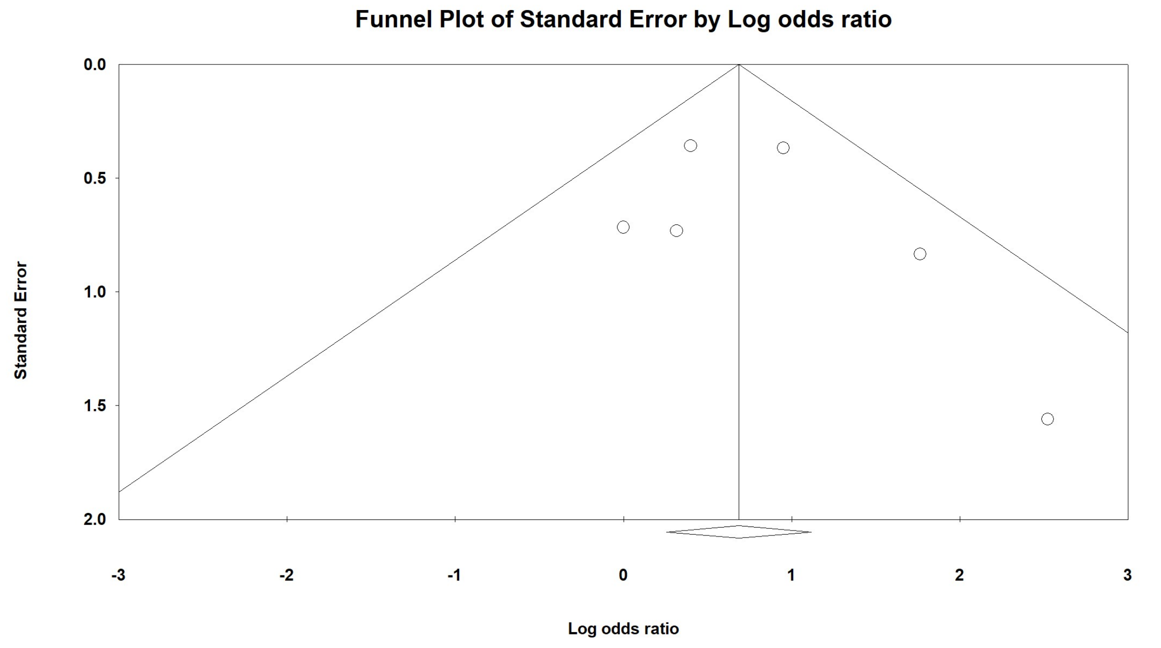

Funnel Plot of Standard Error by Log odds ratio
Standard Error
0.0
0.5
1.0
1.5
2.0
-3
-2
-1
0
1
2
3
Log odds ratio

## Egger's regression intercept

| | |
|---|---|
| Intercept | 0.84759 |
| Standard error | 1.02612 |
| 95% lower limit (2-tailed) | -2.00136 |
| 95% upper limit (2-tailed) | 3.69655 |
| t-value | 0.82602 |
| df | 4.00000 |
| P-value (1-tailed) | 0.22761 |
| P-value (2-tailed) | 0.45521 |

## Begg and Mazumdar rank correlation

| | |
|---|---|
| **Kendall's S statistic (P-Q)** | 9.00000 |

**Kendall's tau without continuity correction**

| | |
|---|---|
| Tau | 0.60000 |
| z-value for tau | 1.69081 |
| P-value (1-tailed) | 0.04544 |
| P-value (2-tailed) | 0.09087 |

**Kendall's tau with continuity correction**

| | |
|---|---|
| Tau | 0.53333 |
| z-value for tau | 1.50294 |
| P-value (1-tailed) | 0.06643 |
| P-value (2-tailed) | 0.13285 |
