# Supplementary material for: Meta-analysis reveals apolipoprotein ε4 confers higher susceptibility to Parkinson’s disease dementia in Asian populations
Source: Front Aging Neurosci. 2026 Mar 10;18:1737073. doi: 10.3389/fnagi.2026.1737073 (PMC13008656; doi:10.3389/fnagi.2026.1737073)
Supplement: Supplementary file 2 [file Data_Sheet_2.pdf]

Supplementary Table S1. Database-Specific Literature Search Strategies

Database	Search Strategy
**PubMed**	(“Parkinson Disease”[MeSH] OR “Parkinson’s disease” OR PD) AND (“Dementia”[MeSH] OR dementia OR “Parkinson disease dementia” OR PDD) AND (“Apolipoprotein E”[MeSH] OR APOE OR “APOE polymorphism” OR ε4 OR ε3 OR ε2)
**Embase**	(‘parkinson disease’/exp OR ‘parkinson* disease’ OR PD) AND (‘dementia’/exp OR dementia OR ‘parkinson disease dementia’ OR PDD) AND (‘apolipoprotein e’/exp OR APOE OR ‘APOE polymorphism’ OR epsilon 4 OR epsilon 3 OR epsilon 2)
**Web of Science**	TS = (Parkinson* disease OR PD) AND TS = (dementia OR “Parkinson disease dementia” OR PDD) AND TS = (APOE OR “apolipoprotein E” OR polymorphism* OR ε4 OR ε3 OR ε2)
**Cochrane Library**	(Parkinson* disease OR PD) AND (dementia OR “Parkinson disease dementia” OR PDD) AND (APOE OR “apolipoprotein E” OR polymorphism*)
**Google Scholar**	“Parkinson disease dementia” OR PDD AND APOE OR “apolipoprotein E” AND polymorphism OR ε4 OR ε3 OR ε2
**WanFang**	(帕金森病 OR 帕金森) AND (痴呆 OR 帕金森病痴呆) AND (载脂蛋白E OR APOE OR 基因多态性)
**CNKI**	(帕金森病) AND (痴呆 OR 帕金森病痴呆) AND (载脂蛋白E OR APOE OR 多态性)
